# The histological and histometrical effects of *Urtica dioica* extract on rat’s prostate hyperplasia 

**Published:** 2015-03-15

**Authors:** Hamid Reza Moradi, Naeem Erfani Majd, Saleh Esmaeilzadeh, Sayed Reza Fatemi Tabatabaei

**Affiliations:** 1 *Department of Basic Sciences, Faculty of Veterinary Medicine, Shahid Chamran University of Ahvaz, Ahvaz, Iran; *; 2 *Department of Pathobiology, Faculty of Veterinary Medicine, Shahid Chamran University of Ahvaz, Ahvaz, Iran.*

**Keywords:** Histometry, Hyperplasia, Prostate, Rat, *Urtica dioica*

## Abstract

Benign prostatic hyperplasia (BPH) is a common disease in human that gradual overgrowth of the prostate gland leads to impinge on the urethra with impairment in urinary function. Numerous plants improve uncontrolled growth of the prostate gland and improve urinary tract symptoms associated with BPH. In this study, 25 healthy adult male Wistar rats were divided randomly in five groups: G1 (Control group) received ordinary feed without any treatment, G2 received 10 mg kg^-1 ^testosterone subcutaneously, G3 received 50 mg kg^-1 ^nettle root extract orally, G4 received 50 mg kg^-1 ^nettle root extract orally and 10 mg kg^-1 ^testosterone, G5 received 10 mg kg^-1 ^almond oil (Almond oil was used as testosterone solvent) subcutaneously. After six weeks, volume and weight of each lobe were measured and samples were taken. The 5 to 6 µm thickness sections were made using paraffin embedding method and stained by hematoxylin and eosin and periodic acid-Schiff. The results showed that prostate volume and ratio of prostate to body weight were increased significantly in the testosterone. Histological and histometrical results showed that dorsal and lateral type 1 and 2 lobes were not changed significantly but the ventral and anterior lobes have changed significantly. Over all, the nettle root could prevent from some of prostatic hyperplasia effects, so that percentage of folded alveoli in ventral lobe reduced insignificantly.

## Introduction

Prostate gland plays an important role in active reproductive period of males. Extensive research has been done on the relationship between structure and function in various ways in number of animals and humans.^1^ The prostate is an exocrine gland which its function and development are androgen-dependent.^2^ Androgens play an important role in developing prostate disorders including prostate cancer and benign prostatic hyper-plasia.^3^ Benign prostatic hyperplasia is the most common male genital tract problems that prevalent with aging.^4^ So far, many researches have been done in relation to the prevention and treatment of this disease. Rat prostate gland has different lobes which their structures are different significantly.^5^ Therefore, there are different reports about response of the gland to testosterone. Mohammady *et al*. have reported that ventral lobe of rat prostate has not responded to induction of hyperplasia using of testosterone.^6^ But other researchers have confirmed hyperplasia induced by testosterone in the ventral lobe of rat’s prostate gland.^7^^,^^8^ Numerous medicinal plants have proven to improve uncontrolled growth of the prostate gland and improve urinary tract symptoms associated with benign prostatic hyperplasia.^7^ Nettle is one of the herbs which have wide therapeutic properties and it is used for treatment of prostatic hyperplasia widely. It has been reported that clinical symptoms of prostatic hyper-plasia was improved with Nettle.^9^ Nettle root extract is inhibitor of the aromatase enzyme in prostate tissue. Aromatase converts testosterone into estrogen and estrogen hormone is associated with prostate disease most commonly.^10^ Lygans are one of the components of nettle root extract which prevent binding of androgens to sex hormone binding globulin in benign prostatic hyperplasia,^11^ but these mechanisms of action are not clear completely. The present study was purposed to determine histomorphometrical changes of rat’s prostate gland lobes following administration of testosterone and nettle root extract. 

## Materials and Methods

In this study, 25 adult male Wistar rats with average weight of 290 ± 20 g, and 3.5 to 4 months of age were used. Animals were maintained in order to adapt to the environmental conditions for one week. Testosterone (Organon, Cidico, Egypt), nettle extract root (Barij Essence Co., Kashan, Iran), almond oil (Kimiagar Toos Co., Mashhad, Iran) and testosterone measurement kit (DRG Instruments, Marburg, Germany) were used. Rats were divided randomly into five groups five animals each: G1 (control group) rats were fed on ordinary without any treatment; G2 received 10 mg kg^-1 ^testosterone subcutaneously daily; G3 received 50 mg kg^-1 ^nettle root extract orally (by gavage) daily; G4 received 10 mg kg^-1 ^testosterone along with 50 mg kg^-1 ^nettle root extract daily; G5 received 10 mg kg^-1 ^almond oil subcutaneously (Almond oil was used as testosterone solvent). The experiment had continued for six weeks. After this period, the rats were sacrificed using chloroform and after weighting, blood samples were taken for measuring of testosterone by enzyme-linked immunosorbent assay (ELISA). Then, abdominal cavity was explored and samples were taken from different lobes of rat's prostate gland. Volume and weight of the prostate gland were measured for each group. After removing the attachments of the prostate to seminal vesicles, bladder and urethra, the prostate volume was measured by water displacement method in a graduated cylinders containing distilled water. The 5 to 6 µm thickness sections were made by paraffin embedding method and were stained by hematoxylin and eosin (H & E) and periodic acid-Schiff (PAS). Periodic acid-Schiff was used to indicate the cell glycoprotein contents. The histomorphometrical studies were done using digital Dino-Lite lens and Dino-capture 1 software (AnMo Electronics Corp., New Taipei City, Taiwan). Percentage of parenchyma to stroma was measured by graduated ocular. Data are expressed as mean ± standard deviation. One way analysis of variance and post Hoc of Tukey was performed on the data. Differences between groups were considered to be significant in *p *< 0.05.

## Results

**Fig. 1:**
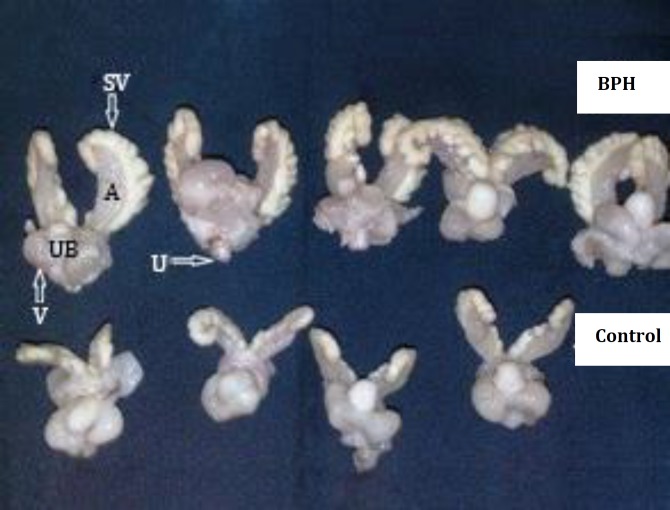
Macroscopical view of rats’ prostate in control and testosterone groups. The prostate enlargement by testosterone is observable. **SV:** Seminal Vesicle, **A:** anterior lobe, **V:** Ventral lobe **UB:** Urinary bladder, **U** **:**Urethra.

**Macroscopic findings.** Table 1 shows the ratio of prostate to body weight and prostate volume were increased significantly in testosterone group compared to the control, nettle and almond oil groups (*p *= 0.000). Prostate volume in testosterone plus nettle group compared to testosterone group were decreased significantly (*p* = 0.028) but the ratio of prostate to body weight were decreased insignificantly (Fig. 1). Prescribed nettle alone lead to a significant reduction in prostate volume compared to the control group (*p *= 0.028). Over all, the volume of prostate and ratio of prostate to body weight in testosterone and nettle groups were maximum and minimum, respectively. Also, the statistical analysis data showed that there was no significant difference in rat weight of different groups. Thus, administration of this hormone, almond oil and nettle root extract did not have significant effect on body weight.

**Table 1:** Macroscopic parameters of prostate in different groups (Mean ± SD).

**Groups**	**Body weight (g)**	**Absolute weight (g)**	**Prostate weight/Body weight ** ***×*** ** 10** ^-3^	**Prostate volume (mL)**
**Control **	316.17 ± 20.86	2.63 ± 0.20^bc^	7.70 ± 0.44^b^	3.20 ± 0.45^ab^
**Testosterone **	296.14 ± 12.87	3.76 ± 0.46^a^	12.02 ± 1.03^a^	4.60 ± 0.65^a^
**Nettle **	310.89 ± 25.15	2.59 ± 0.27^bc^	7.80 ± 0.30^b^	2.30 ± 0.27^c^
**Testosterone + Nettle **	289.20 ± 6.18	3.24 ± 0.08^ab^	11.46 ± 0.96^a^	3.70 ± 0.27^ab^
**Almond oil **	313.51 ± 23.98	2.44 ± 0.54^c^	7.15 ± 1.00^b^	2.40 ± 0.42^c^

abc Different letters in each column indicate significant differences (*p* < 0.05).

**Fig. 2:**
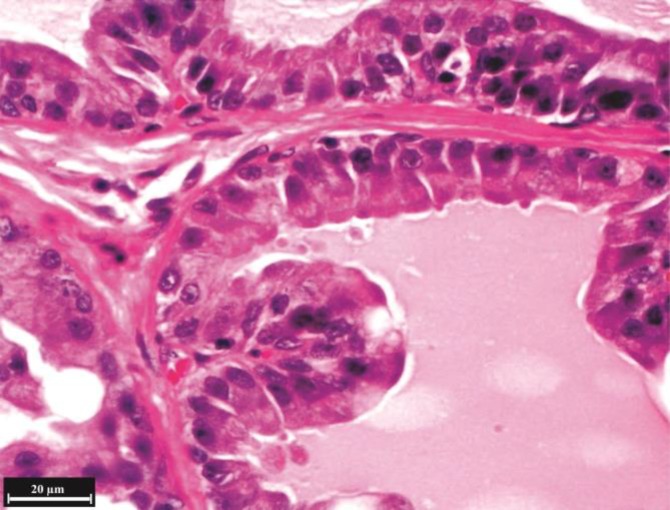
Histological structure of ventral lobe in the testosterone group, (H & E). The increase of alveoli folding and height of the secretory epithelium are considerable.

**Fig. 3:**
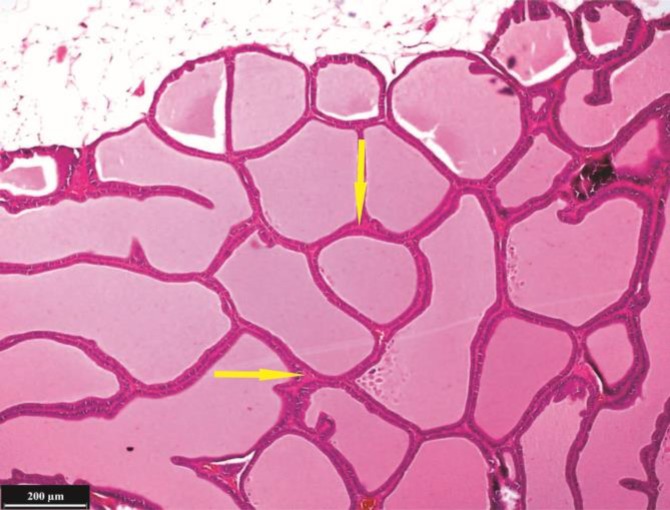
Histological structure of ventral lobe in the control group, (H & E). The high densities of secretory alveoli are considerable. Connective tissue between the secretory alveoli (arrows).

**Fig. 4:**
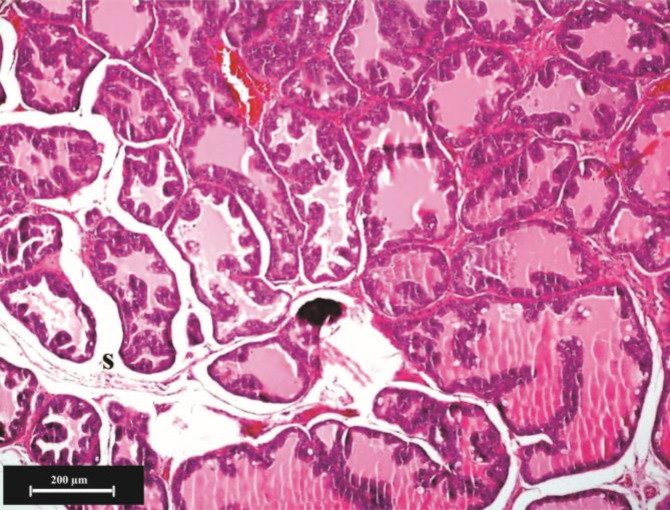
Histological structure of ventral lobe in the testosterone group, (H & E). Increase in alveoli folding is considerable. Stromal connective tissue (**S**) was increased between the secretory alveoli.

**Fig. 5:**
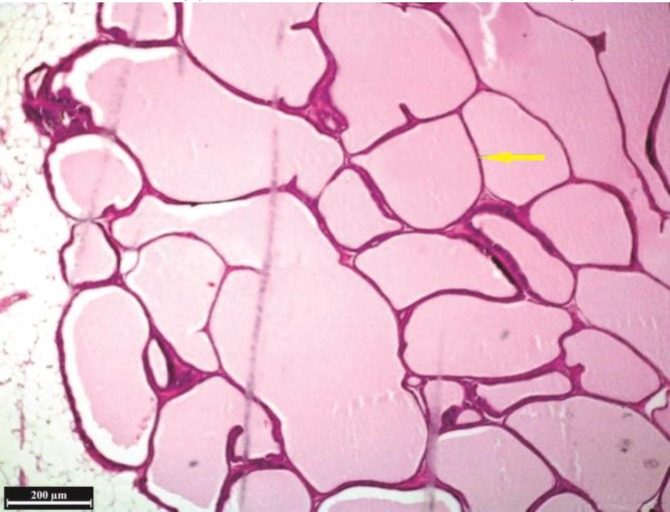
Histological structure of ventral lobe in the nettle group, (H & E). The expanding of secretory alveoli and their thin-walled are considerable (arrow).

**Fig. 6:**
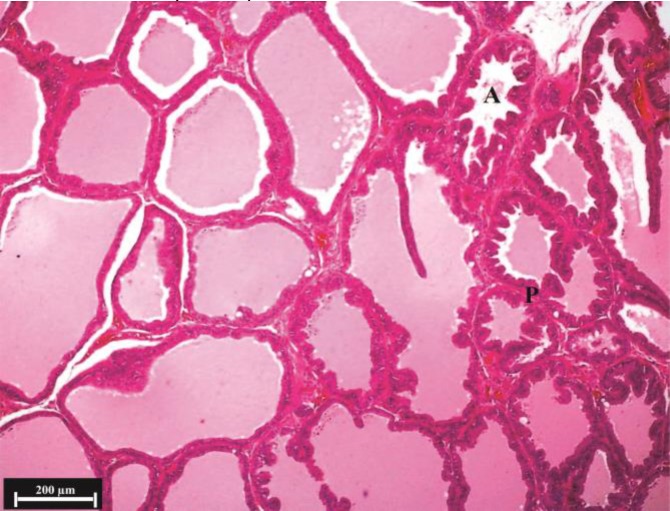
Histological structure of ventral lobe in the testosterone + nettle group, (H & E). Decrease in folded secretory alveoli (**A**) in the periphery of a lobe (**P**) is considerable.

**Microscopic findings: Ventral lobe. **Testosterone resulted in growth and proliferation of ventral prostate epithelium severely and secretory cells were long cylindrical cells and active in secretion (Fig. 2). The secretory cells height (*p *= 0.000), number (in scale of 100 µm epithelium length) (*p *= 0.013) and folding in wall of alveolus (*p *= 0.000) were significantly increased in the testosterone group (Figs. 3 and 4). Percentage of parenchyma to stroma of ventral lobe was decreased significantly in the testosterone group compared to the control (*p* = 0.029) and almond oil groups (*p* = 0.048). Nettle lead to folding reduction and decreased alveolar wall thickness of the ventral lobe compared to control and almond oil groups (Fig. 5). Diameter of secretory alveoli was the lowest in nettle group than other groups. Secretory cells were low to high cuboidal in the nettle group and nucleus was heterochromatin and located in the basal cytoplasm. The percentage of folded alveoli and their diameters were decreased in the ventral lobe of nettle plus testosterone group compared to testosterone group, but it was not the same as control group (Fig. 6). The secretory cells height and number were increased significantly in testosterone plus nettle group compared to control and almond oil groups (*p* = 0.000), (Table 2). 

**Table 2:** The characteristic changes of the rat prostate ventral lobe in the experimental groups (Mean ± SD).

**Groups **	**Epithelial ** **thickness** **(µm)**	**Number of cells** **in scale ** **100 µm**	**Percentage of ** **folded secretory alveolar units**	**Percentage of parenchyma to stroma**	**Diameter of alveolar units ** **(µm)**
**Control **	14.20 ± 0.81^b^	12.85 ± 0.84^b^	38.02 ± 3.94^b^	88.00 ± 3.60^b^	224.64 ± 8.77^ab^
**Testosterone **	23.04 ± 2.73^a^	17.00 ± 3.06^a^	71.99 ± 14.59^a^	77.00 ± 5.70^a^	240.78 ± 32.62^a^
**Nettle **	12.82 ± 2.68^b^	12.25 ± 0.88^b^	27.95 ± 8.24^c^	87.20 ± 7.00^b^	188.01 ± 20.04^b^
**Testosterone + Nettle **	23.37 ± 2.97^a^	18.05 ± 1.68^a^	55.70 ± 12.60^ab^	80.80 ± 6.10^ab^	220.3 ± 24.82^ab^
**Almond oil **	12.14 ± 0.50^b^	13/00 ± 1.26^b^	31.14 ± 8.33^c^	89.20 ± 3.35^b^	253.42 ± 30.54^a^

abc Different letters in each column indicate significant differences (*p* < 0.05).

**Fig. 7.**
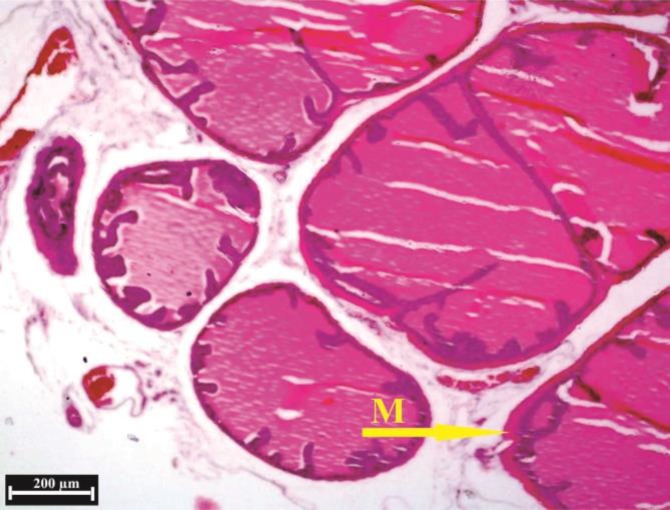
Histological structure of anterior lobe in the control group, (H & E). The alveoli epithelium of anterior lobe have numerous folds normally. Muscle layer surrounding the secretory alveoli (**M**).

**Fig. 8:**
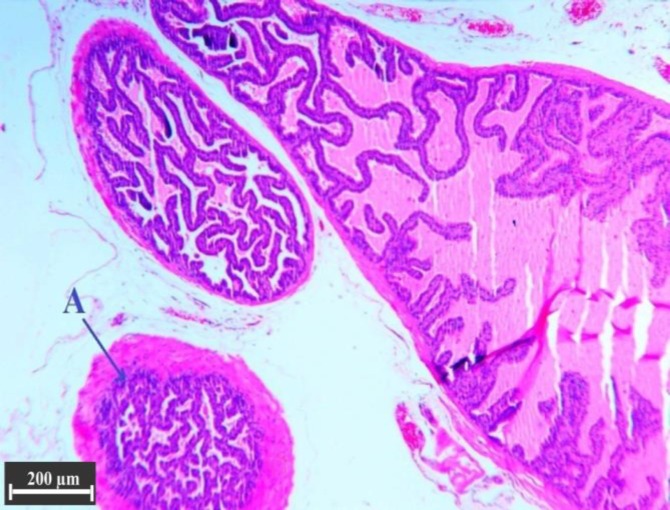
Histological structure of anterior lobe in the testosterone group, (H & E). The number and length of the folds in alveoli are increased, therefore the alveolar lumen (**A**) is filled and they connected together. Thick muscle layers surrounding the secretory alveoli are considerable.

**Microscopic findings: Anterior lobe.** Normally, epithelium in the anterior lobe had a lot of folds (Fig. 7). However, under the influence of testosterone, height and number of epithelium folds were grown, thus, they filled alveolar cavity. Muscle cells were increased in several layers around alveoli compared to control group (Fig. 8). Proliferations of secretory cells were seen in testosterone group. Also, the number of secretory cells in 100 µm length of epithelium were increased significantly in the testosterone group (*p* = 0.045). Secretory cells in different prostate lobes was PAS positive reaction in normal rat prostate, but staining intensity was more in the anterior lobes. Hyperplasia was observed in the secretory alveoli of anterior lob. Folds were expanded in the lumen of secretory alveoli and the numbers of folded alveoli were increased. 

**Table 3:** The characteristic changes in the rat prostate anterior lobe in the experimental groups (Mean ± SD).

**Groups **	**Epithelial ** **thickness** **(µm)**	**Number of cells** **in scale ** **100 µm**	**Percentage of ** **folded secretory alveolar units**	**Percentage of parenchyma to stroma**	**Diameter of alveolar units ** **(µm)**
**Control **	16.85 ± 2.30	11.40 ± 0.98^b^	100	75.40 ± 5.40^b^	443.96 ± 140.72^ab^
**Testosterone **	17.66 ± 2.62	13.60 ± 1.44^a^	100	84.20 ± 2.60^a^	618.33 ± 191.23^a^
**Nettle **	14.27 ± 1.13	12.45 ± 1.02^ab^	100	82.00 ± 5.70^ab^	366.98 ± 82.50^b^
**Testosterone + Nettle **	15.72 ± 1.28	12.95 ± 0.94^ab^	100	82.40 ± 2.70^ab^	414.71 ± 8.05^ab^
**Almond oil **	14.62 ± 1.28	11.35 ± 1.24^b^	100	75.40 ± 4.60^b^	366.26 ± 47.36^b^

ab Different letters in each column indicate significant differences (*p* < 0.05).

**Table 4:** The characteristic changes in the rat prostate lateral type 1 lobe in the experimental groups (Mean ± SD).

**Groups **	**Epithelial ** **thickness** **(µm)**	**Number of cells** **in scale ** **100 µm**	**Percentage of ** **folded secretory alveolar units**	**Percentage of parenchyma to stroma**	**Diameter of alveolar units ** **(µm)**
**Control **	14.48 ± 2.94	11.25 ± 0.40^a^	43.48 ± 5.36	74.20 ± 2.05^ab^	214.07 ± 23.07^a^
**Testosterone **	16.78 ± 1.77	10.55 ± 0.54^ab^	43.55 ± 4.16	77.00 ± 1.87^ab^	209.74 ± 31.24^ab^
**Nettle **	15.62 ± 1.50	10.15 ± 1.05^ab^	53.68 ± 3.17	70.60 ± 1.95^b^	168.90 ± 19.76^b^
**Testosterone + Nettle **	16.57 ± 1.15	9.85 ± 0.63^b^	51.11 ± 5.93	78.20 ± 5.97^a^	203.74 ± 16.67^ab^
**Almond oil **	13.46 ± 0.64	10.00 ± 0.75^ab^	44.85 ± 9.2	75.40 ± 3.29^ab^	199.51 ± 19.13^ab^

ab Different letters in each column indicate significant differences (*p* < 0.05).

**Table 5:** The characteristic changes in the rat prostate lateral type 2 lobe in the experimental groups (Mean ± SD).

**Groups **	**Epithelial ** **thickness** **(µm)**	**Number of cells** **in scale ** **100 µm**	**Percentage of ** **folded secretory alveolar units**	**Percentage of parenchyma to stroma**	**Diameter of alveolar units ** **(µm)**
**Control **	13.98 ± 1.86	11.40 ± 0.52	49.54 ± 4.01	74.40 ± 2.19	189.94 ± 22.34^ab^
**Testosterone **	17.19 ± 1.48	11.15 ± 0.78	43.13 ± 3.07	74.80 ± 5.63	220.53 ± 26.39^a^
**Nettle **	15.03 ± 2.78	10.70 ± 0.97	48.41 ± 2.65	80.60 ± 2.97	177.95 ± 13.82^b^
**Testosterone + Nettle **	16.28 ± 1.24	10.15 ± 0.45	48.77 ± 4.09	80.40 ± 4.67	197.85 ± 24.51^ab^
**Almond oil **	13.93 ± 0.96	10.20 ± 0.82	41.84 ± 4.84	75.00 ± 4.47	175.78 ± 11.72^a^

ab Different letters indicate significant differences (*p* < 0.05).

**Table 6:** The characteristic changes in the rat prostate dorsal lobe in the experimental groups (Mean ± SD).

**Groups **	**Epithelial ** **thickness** **(µm)**	**Number of cells** **in scale ** **100 µm**	**Percentage of ** **folded secretory alveolar units**	**Percentage of parenchyma to stroma**	**Diameter of alveolar units ** **(µm)**
**Control **	13.80 ± 2.82^ab^	10.30 ± 0.82	48.86 ± 9.49	69.40 ± 4.62^b^	195.11 ± 53.52^ab^
**Testosterone **	16.20 ± 1.24^a^	12.30 ± 2.26	60.92 ± 6.71	72.00 ± 2.55^ab^	249.55 ± 33.71^a^
**Nettle **	14.01 ± 1.23^ab^	10.50 ± 0.91	58.38 ± 7.85	77.20 ± 3.96^a^	168.94 ± 32.29^b^
**Testosterone + Nettle **	16.98 ± 1.54^a^	10.85 ± 0.58	60.06 ± 5.78	69.80 ± 2.64^ab^	235.78 ± 32.06^ab^
**Almond oil **	12.95 ± 0.94^b^	58.72 ± 11.10	58.72 ± 11.10	72.20 ± 5.67^ab^	174.58 ± 33.16^b^

ab Different letters in each column indicate significant differences (*p* < 0.05).

The number and height of secretory cells of anterior lobe were decreased in nettle plus testosterone group. Epithelial thickness in the nettle group was decreased insignificantly compared to the control group. Percentage of parenchyma to stroma in the anterior lobe in testosterone group was increased significantly (*p* = 0.036), (Table 3).

Histometrical results showed that percentage of parenchyma of dorsal lobe was increased in nettle group compared to control group significantly (*p* = 0.046). Percentage of parenchyma of lateral type 1 lobe was decreased in nettle group compared to nettle plus testosterone group significantly (*p* = 0.016). Diameter of alveoli of dorsal lobe had significant increase in testosterone group than almond oil group (*p* = 0.038). Diameter of alveoli of lateral type 1 lobe had significant decrease in nettle group compared to control group (*p* = 0.035). Diameter of alveoli of lateral type 2 lobe had significant decrease in testosterone group than almond oil group (*p* = 0.020), (Tables 4, 5 and 6).

**Fig. 9:**
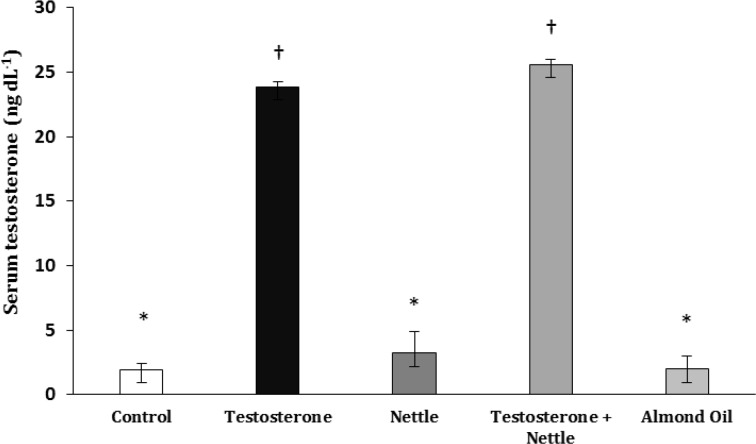
Serum testosterone of the experimental groups (Mean ± SD). *^†^ Different symbols indicate significant differences among the experimental groups (*p *> 0.05).

**Serum testosterone. **The serum testosterone levels was increased significantly in both testosterone and testosterone plus nettle groups compared to control, almond oil and nettle groups (*p* = 0.000). The serum testosterone levels did not have a significant difference between testosterone and testosterone plus nettle groups. The serum testosterone level was increased insignificantly in the nettle group compared to the control group (Fig. 9). 

## Discussion

 In the present study, the BPH which induced by testosterone was characterized by parenchyma and stroma changes and enlargement of rat’s prostate. According to Porpiglia *et al*. and Pais studies which they reported that prostate epithelial cells number and stroma were increased in BPH.^12^^,^^13^ Eaton has reported that the androgens are interacting with connective tissue cells of the prostate gland.^14^ Recent studies showed that the rat ventral prostate lobe is androgen-dependent but the dorsal and lateral lobes are not dependent on it.^15^^-^^18^ The fundamental biological differences exist between the different lobes of the rat prostate.^19^ On the other hand, Ahonen *et al*. reported that hyperplasia in the dorsal and lateral lobes of rat prostate gland caused by hyper-prolactinemia.^20^ Banerjee *et al*. has reported that the dorsal and lateral lobes are more sensitive to androgens in aged (24-month-old) Brown Norway rats than young (6-month-old) rats.^21^ Therefore, the weight and protein content are more in the dorsal and lateral lobes of 24-month-old rats compared to the 6-month-old rats. Many of studies have concentrated on the ventral, dorsal and lateral lobes of prostate rat but the anterior lobe was less considered and it was considered as separated structure from the rat prostate.^19^^,^^22^^,^^23^ According to results of the present study, ventral and anterior lobes were more sensitive and showed more histomorphological changes than dorsal and lateral lobes. Cunha *et al*. have reported that the anterior lobe of rat prostate is similar to the central zone of human prostate.^2^

We also observed that the oral administration of nettle root extract (50 mg kg^-1^) leads to decrease the number of folds in secretory alveolar epithelium and height of folds in the anterior and ventral lobes tissue. Also, secretory cells were altered from cuboidal to squamous which indicate the decrease of secretory activity. These findings indicate that nettle root extract may be effective for BPH. The histological changes following administration of nettle are similar to other medicinal plants such as *Serenoa repens *and Echinacea. Nettle root extract polysaccharides and lectins particularly are important in the prostate gland disorders.^24^ Nettle root contains biologically active compounds and its effect has been attributed to more than one class of chemicals. Therefore, it is reported that, the lignan, sterols, flavonoids, poly-saccharides, lectins, and fatty acids are responsible for the pharmacological effects of nettle.^11^^,^^25^

Serum testosterone levels were more significant in the nettle root extract and testosterone plus nettle groups than other groups. The change of testosterone serum levels confirmed the nettle root extract effects. These results confirmed Chrubasik *et al*.^26^ They showed that the nettle root extract blocked 5α–reductase enzyme activity and prevented the conversion of testosterone to dihydro-testosterone (DHT). Therefore, the serum testosterone level was increased by nettle, thus blood DHT levels were decreased and cell proliferation in the prostate tissue was reduced subsequently.^26^ Results of present study showed that nettle had similar effect of finasteride which is used for treatment of prostatic hyperplasia widely.^27^

According to the results of the present study, the anterior and ventral lobes were more sensitive to hormonal response than the dorsal and lateral (1 and 2) lobes and they were distinguishable and separable from other lobs. Therefore, the anterior and ventral lobes are more suitable for prostate investigations. Finally, it was concluded that prostatic hyperplasia could be reduced by oral administration of nettle root extract and it has protective effects on prostatic hyperplasia.
